# Supplementary material for: The interactome of intact mitochondria by cross-linking mass spectrometry provides evidence for coexisting respiratory supercomplexes
Source: Mol Cell Proteomics. 2017 Dec 8;17(2):216–32. doi: 10.1074/mcp.RA117.000470 (PMC5795388; doi:10.1074/mcp.RA117.000470)
Supplement: Supplemental Data [file supp_RA117.000470_133922_0_supp_23978_fzffwf.zip › spectra_annotation/mito_spectra_annotation/476-1-4-1-11-1.pdf]

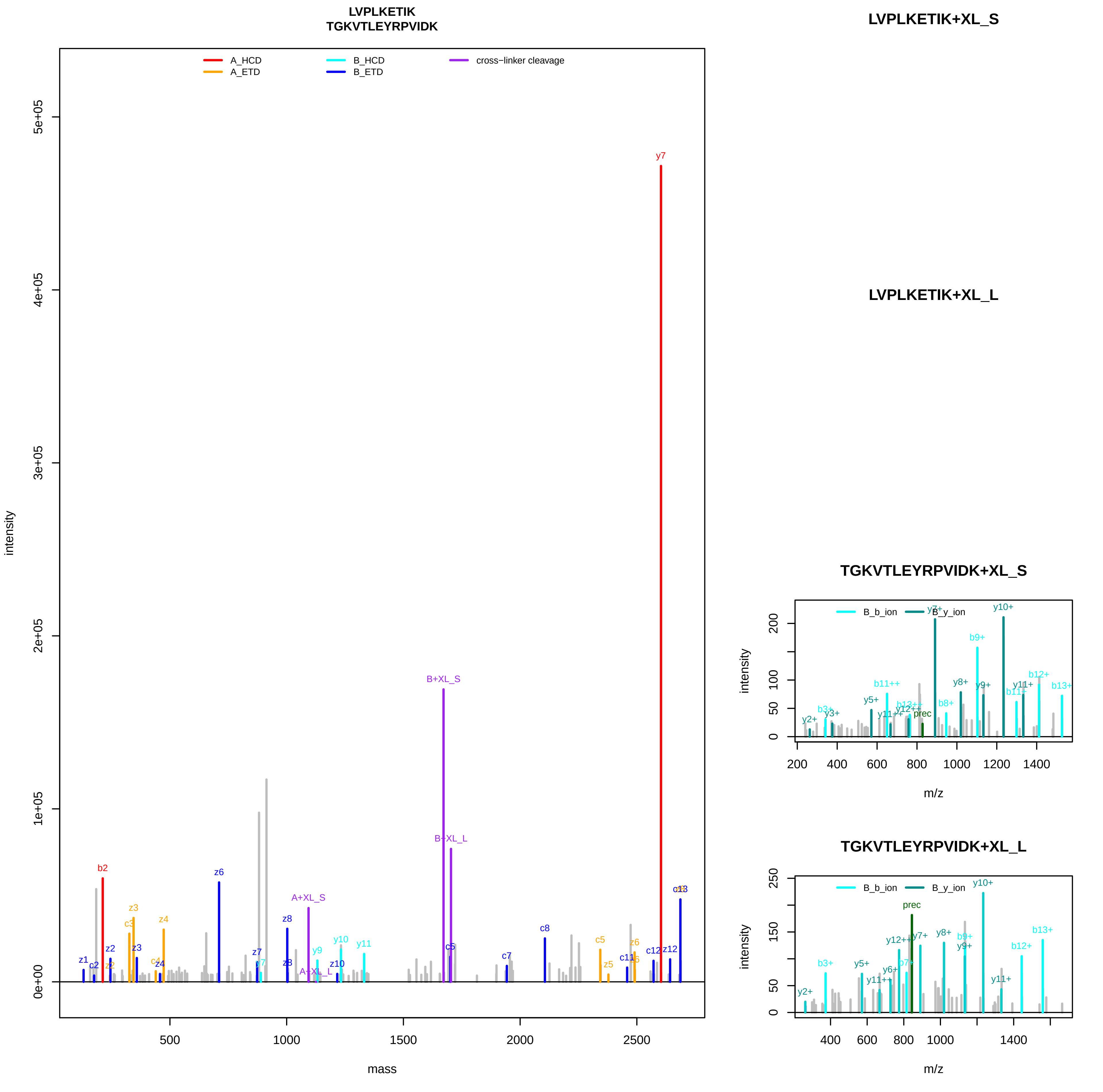

LVPLKETIK
TGKVTLEYRPVIDK
A_HCD
A_ETD
B_HCD
B_ETD
cross−linker cleavage
intensity
mass
0e+00
1e+05
2e+05
3e+05
4e+05
5e+05
500
1000
1500
2000
2500
y7
B+XL_S
B+XL_L
A+XL_S
A+XL_L
b2
z6
z8
c8
c13
z12
c12
c11
c5
z6
z5
c7
y10
y11
y9
z10
z7
y7
z3
c3
z4
c4
z2
z1
c2
LVPLKETIK+XL_S
LVPLKETIK+XL_L
TGKVTLEYRPVIDK+XL_S
B_b_ion
B_y_ion
intensity
m/z
0
50
100
150
200
200
400
600
800
1000
1200
1400
y10+
y7+
b9+
b12+
b13+
y8+
y9+
y11+
b11+
b11++
y5+
b8+
prec
y12++
b13++
y11++
b3+
y3+
y2+
TGKVTLEYRPVIDK+XL_L
B_b_ion
B_y_ion
intensity
m/z
0
50
100
150
200
250
400
600
800
1000
1400
y10+
prec
b13+
b12+
y8+
b9+
y9+
y7+
y12++
y5+
b3+
y6+
b7+
y11++
y11+
y2+
